# Xanthine oxidase inhibition alleviates the cardiac complications of insulin resistance: effect on low grade inflammation and the angiotensin system

**DOI:** 10.1186/s12967-015-0445-9

**Published:** 2015-03-06

**Authors:** Hany M El-Bassossy, Malcolm L Watson

**Affiliations:** Department of Pharmacology and Toxicology, Faculty of Pharmacy, King Abdulaziz University, Jeddah, Kingdom of Saudi Arabia; Department of Pharmacology and Toxicology, Faculty of Pharmacy, Zagazig University, Zagazig, Egypt; Department of Pharmacy and Pharmacology, University of Bath, Bath, UK

**Keywords:** Metabolic syndrome, Heart, Hypertension, Xanthine oxidase, Allopurinol

## Abstract

**Background:**

We have previously shown that hyperuricemia plays an important role in the vascular complications of insulin resistance (IR). Here we investigated the effect of xanthine oxidase (XO) inhibition on the cardiac complications of IR.

**Methods:**

IR was induced in rats by a high fructose high fat diet for 12 weeks. Allopurinol, a standard XO inhibitor, was administered in the last 4 weeks before cardiac hemodynamics and electrocardiography, serum glucose, insulin, tumor necrosis factor alpha (TNFα), 8-isoprostane, uric acid, lactate dehydrogenase (LDH) and XO activity were measured. Expression of cardiac angiotensin II (AngII) and angiotensin receptor 1 (AT_1_) were assessed by immunofluorescence.

**Results:**

IR animals had significant hyperuricemia which was inhibited by allopurinol administration. IR was associated with impaired ventricular relaxation (reflected by a decreased diastolic pressure increment and prolonged diastolic duration) and XO inhibition greatly attenuated impaired relaxation. IR was accompanied by cardiac ischemia (reflected by increased QTc and T peak trend intervals) while XO inhibition alleviated the ECG abnormalities. When subjected to isoproterenol-induced ischemia, IR hearts were less resistant (reflected by larger ST height depression and higher LDH level) while XO inhibition alleviated the accompanying ischemia. In addition, XO inhibition prevented the elevation of serum 8-isoprostane and TNFα, and blocked elevated AngII and AT_1_ receptor expression in the heart tissue of IR animals. However, XO inhibition did not affect the developed hyperinsulinemia or dyslipidemia.

**Conclusions:**

XO inhibition alleviates cardiac ischemia and impaired relaxation in IR through the inhibition of low grade inflammation and the angiotensin system.

## Background

Insulin resistance (IR) is a key component of metabolic syndrome (MetS). The pathogenesis of the syndrome includes multiple organs involved in the cardiovascular system [1]. The prevalence of MetS in the US in 2002 is estimated at 24% while in the elderly (over 60 years) the prevalence is 44%, based upon the data from the National Health and Nutrition Examination Survey [2]. This is correlated with the dramatic increase of fructose intake in the last hundred years. Unlike other sugars, fructose rapidly causes ATP depletion that results in nucleotide turnover and finally generation of uric acid [3].

Although hyperuricemia is condition that frequently accompanies MetS, its role as a risk factor for cardiovascular complications in MetS is debated. For decades, hyperuricemia was considered as a simple result of renal dysfunction and not as a true mediator but in the late 1990s researchers started to reevaluate hyperuricemia as a causal risk factor for cardiovascular disease [4]. We have previously shown that direct *in vitro* incubation of normal isolated aorta with uric acid at the same concentration observed in IR animals impairs endothelial dependent relaxation [5].

In addition to uric acid production, the xanthine oxidase (XO) enzyme generates reactive oxygen species (ROS) through catalysing oxidation of hypoxanthine to xanthine and then to uric acid. XO-derived reactive oxygen species (ROS) contribute to many pathologic conditions characterized by ischemia or inflammation [6]. Allopurinol is a standard inhibitor of XO. Although this agent has been widely used for treatment of gout for decades, in the last fifteen years it has attracted attention for potential benefits in cardiovascular disease [7].

Therefore, the aim of the present work was to investigate whether XO represents a realistic target for intervention in the context of protection of cardiovascular dysfunction in IR and to suggest the possible mechanism of action for protection. Data presented from this animal study indicate potential new therapeutic strategies for the treatment of human disease.

## Methods

### Animals

The study is reported in accordance with the Animals in Research: Reporting In Vivo Experiments (ARRIVE) guidelines for research involving animals [8], and the Kingdom of Saudi Arabia Research Bioethics and Regulations, consistent with UK standards of care. Male Wistar rats (6 weeks age; King Abdulaziz University, KSA) were housed (3–4 rats per cage) in clear polypropylene cages and kept under constant environmental conditions with equal light–dark cycle. Rats had free access to commercially available rodent pellet diet and purified water.

### Study overview

The experimental protocol was approved by the Unit of Biomedical Ethics Research Committee, King Abdulaziz University, KSA. Animals were randomly divided into three groups (8 animals each) of control (C), IR or allopurinol-treated insulin resistance (IR-Allo). IR was induced by adding fructose (10%) to drinking water and feeding rats on high fat high salt diet (16% crude protein, 28.2% crude fat, 2.8% crude fibre, 4.8% ash, 3.4% salt; prepared in our laboratories) for 12 weeks. Control animals received water and standard diet (20% crude protein, 4% crude fat, 3.5% crude fibre, 6% Ash, 0.5% salt). Allopurinol (20 mg.kg^−1^.day^−1^) was administered daily in the last 4 weeks by dissolving in drinking water (100–110 mg.l^−1^) depending on daily water consumption [9]. In previous work from our laboratories, we have shown that eight weeks of high fructose administration was necessary for developing significant insulin resistance while another four weeks was needed to produce clear vascular complications [10]. In the present work, allopurinol was given in the last four weeks (after establishment of IR and during the development of vascular complications) in order to test the protective effect of allopurinol against the cardiac complications rather than the IR.

At the end of the study animals were fasted for 8 hours. A tail capillary droplet was used to determine the fasting blood glucose level before rats were anaesthetized by urethane (1.5 g.kg^−1^, i.p.) injection [11]. Then, a microtip catheter was inserted in the left ventricle through an opening in the right carotid artery to continuously record cardiac contractility while cardiac conductivity was determined by surface electrocardiography (ECG). After 15 min recording basal cardiac contractility and conductivity, one ml blood was withdrawn from the vena cava (through a small incision in the lower abdomen), allowed to coagulate for 30 min at 4°C, and then centrifuged (3000 × g, 4°C, 20 min) to separate serum. Serum was divided into aliquots and stored at −20°C for later analysis of insulin, tumor necrosis factor alpha (TNFα), adiponectin, 8-isoprostane, uric acid, triglycerides and XO activity. Rats were then injected with one ml saline to prevent hypovolemia. After another 15 min stabilization, acute cardiac ischemia was induced by isoproterenol (10 μg.kg^−1^) injection through the femoral vein [12]. The cardiac contractility and conductivity were continuously recorded for 30 min after isoproterenol injection. At the end of the experiment another millilitre of venous blood was withdrawn for analysis of serum lactate dehydrogenase (LDH) and animals were killed by an overdose of anaesthetic. The heart was quickly dissected and fixed in 10% neutral buffered formalin for immunofluorescence detection of angiotensin II (AngII), angiotensin receptor type 1 (AT_1_), collagen I and 4-hydroxy-2-noneal Michael adducts (4HNE) in heart cross sections.

### Biochemical analysis

Glucose was determined in tail blood samples with a glucose meter (ACCU-CHEK®, Roche Diagnostics, Mannheim, Germany). Serum insulin level was measured by enzyme-linked immunosorbent assay (ELISA, Millipore, Billerica, MA, USA) using plates coated with anti-rat insulin antibodies. Serum TNFα and adiponectin levels were determined by ELISA using Quantikine® kits (R & D systems, Minneapolis, MN, USA) using rat TNFα or rat adiponectin and antibodies raised against rat TNF-α or rat adiponectin respectively. XO activity was measured in the final serum sample using the BioVision® assay kit (BioVision Incorporated, Milpitas, CA, USA). Serum level of 8-isoprostane was determined by a competitive immunoassay 8-isoprostane EIA® kit (Cayman Chemical Company, Ann Arbor, Michigan, USA). Serum levels of uric acid and triglycerides and LDH activity were determined using the ELITech® assay kit (ELITech, Puteaux, France). Serum level of fructosamine was determined using Quimica Clinica® assay kit (Quimica Clinica, Amposta, Spain).

### Cardiac hemodynamic recording

Invasive real time recording of cardiac hemodynamics was carried out according to the method described in a previous report from our laboratories [13]. Following anaesthesia as described above the animals’ body temperature was maintained at 37°C (measured by a rectal probe) using controlled heating pads. A microtip pressure transducer (SPR-320, Millar Instruments, Houston, TX, USA) was inserted through a small incision into the right carotid artery and advanced into the left ventricle. After 5 min stabilization period, the signals were continuously recorded. The microtip catheter was connected to a Power Lab Data Interface Module connected to a PC running Lab Chart professional software (v8.0, AD Instruments, Bella Vista, Australia) including the BP module. The BP module detects and calculates the left ventricular end ventricular systolic pressure (ESP), left ventricular end-diastolic pressure (EDP), slope of the systolic pressure increment (dP/dt) and slope of the diastolic pressure decrement (−dP/dt), cystolic and diastolic duration, and contractility index.

### Electrocardiogram (ECG) recording

The standard surface ECG was recorded by the Powerlab® system (AD Instruments, Bella Vista, Australia) connected to a PC running LabChart professional software with the ECG module to detect different components of the ECG. The change in the ST segment height was used as the index of anginal severity and the ST segment value was defined as the mean ECG voltage 12 ms after the S wave peak [14], and the change in ST segment value after isoproterenol injection was calculated as the ST depression.

### Immunofluorescence Studies

Immunofluorescence staining of AngII, AT_1_, 4HNE and collagen protein in rat paraffin embedded heart sections (5 μm) was carried out according to the method used in our previous work [15-17]. Fixed heart tissue section slides were deparaffinized in xylene and rehydrated in ethanol and distilled water. Then, perforation was carried out by incubation with methanol at −20°C for 30 min followed by washing with distilled water. Epitopes were retrieved (antigen retrieval) in citrate buffer for 30 min at 95°C followed by washing with phosphate buffered saline (PBS). Slides were then immediately transferred into a humidity chamber. Nonspecific binding sites were blocked (bovine serum albumin in PBS containing 5% normal goat serum, 1% BSA, 0.1% Triton) at room temperature for 1 h. After the blocking, sections were washed (3 x 5 min) with PBS. Heart sections were then incubated with the intended primary antibody diluted in blocking buffer at 4°C overnight. The sections were then washed (3 x 5 min) with PBS followed by incubation with a fluorescent conjugated secondary antibody (diluted 1:200 in blocking buffer) for 1 h in the dark. Then sections were washed (3 x 5 min) with PBS and slides were dried and mounted with ‘ProLong’ mounting media (Life Technologies, Paisley, UK). The slides were stored in dark overnight before examination with a Zeiss LSM 780 confocal microscope (Carl Zeiss, Gottingen, Germany) at 488 and 561 nm excitation and 497–542 and 596–655 nm emission filters). Images were acquired with identical acquisition parameters, with minimum excitation and gain. Quantitative comparisons of image fluorescence were made with Image J software (National Institute of Health, Bethesda, MD, USA). For presentation purposes, the level of the confocal images were equally adjusted after the fluorescence quantifications were carried out on unmanipulated images. Sections treated with the secondary antibody alone did not show specific fluorescence while incubating the primary antibody with the blocking peptide significantly reduced the signal. The primary antibodies used were: rabbit polyclonal anti AngII (1:2000, Phoenix Pharmaceuticals Inc., Burlingame, CA, USA), mouse monoclonal anti AT_1_ receptor (1:133, Abcam, Cambridge, MA, USA), mouse monoclonal anti collagen type I (1:1000, Abcam), rabbit polyclonal anti 4HNE (1:250, Millipore, Billerica, MA, USA). The secondary antibodies used were Alexa Fluor (λex = 488) conjugated goat anti-mouse and Alexa Fluor (λex = 594) conjugated goat anti-rabbit (1:200, Life Technologies, Grand Island, NY, USA).

### Drugs and chemicals

The following drugs and chemicals were used: Allopurinol, urethane (Sigma-Aldrich, St. Louis, MO, USA). Allopurinol was dissolved in distilled water while urethane was dissolved in saline.

### Statistical analysis

Values are expressed as mean ± SEM. Statistical analysis was carried out by analysis of variance (ANOVA) followed by Newman-Keuls’ post hoc test using statistical software (Prism 5, GraphPad, CA, USA).

## Results

### Uric acid level and XO activity

**Figure 1:**
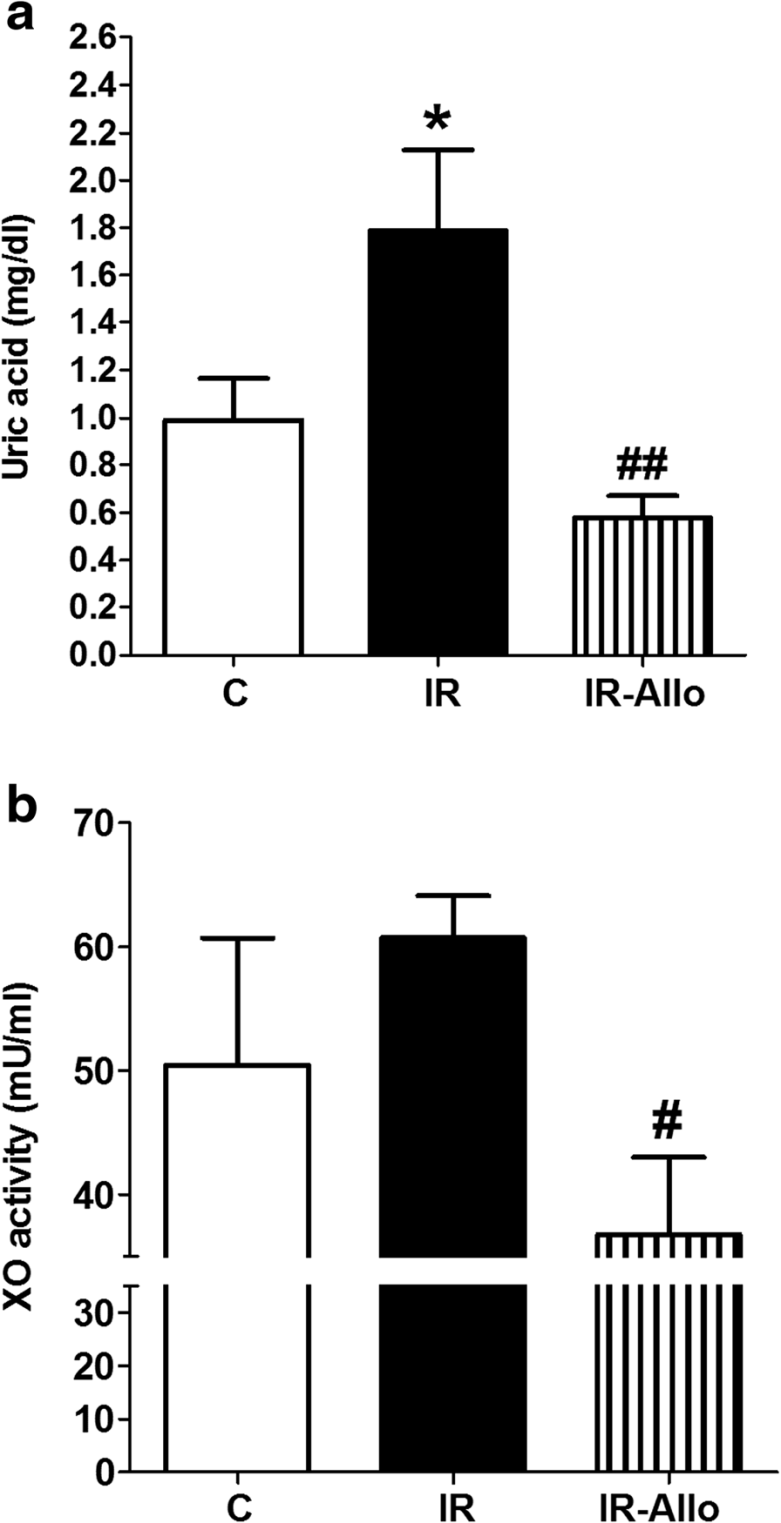
**Effect of allopurinol (Allo) administration (20 mg.kg** ^**−1**^ **.day** ^**−1**^ **) in the last 4 weeks on serum levels of uric acid (a) and xanthine oxidase activity (b) in rats with high fructose high fat (for 12 weeks) -induced insulin resistance (IR).** Data are presented as mean ± standard error of 8 animals in each group. ^*^P < 0.05, compared with the corresponding control group values; ^#^P < 0.05, ^##^P < 0.01, compared with the corresponding IR group values; by one way ANOVA and Newman Keuls’ *post hoc* test.

High fructose high fat diet administration was associated by a significant elevation in serum uric acid level compared to control (p < 0.05, Figure 1a) while allopurinol administration significantly inhibited the developed hyperuricemia (p < 0.01, Figure 1a). The serum XO activity tended to increase in rats fed on high fructose high fat diet while allopurinol administration significantly inhibited serum XO activity compared to the high fructose high fat diet fed group (p < 0.05, Figure 1b).

### Biochemical parameters

There was no difference in the fluid consumption between the three groups. Caloric intake was 552, 742 and 735 Kcal/day/kg in the control, IR, and IR-Allo groups respectively.

Insulin resistance (IR) development after twelve weeks feeding on high fructose and high fat diet was evidenced by the significantly higher weight gain (p < 0.01), glucose (p < 0.001), insulin (p < 0.05) and triglyceride (p < 0.05) levels in the IR group than those in the control group. XO enzyme inhibition by allopurinol administration in the last four weeks did not significantly affect the exaggerated weight gain, the developed hyperglycemia, hyperinsulinemia or hypertriglyceridemia associated with the high fructose and high fat diet (Table 1).

**Table 1:** **Effect of xanthine oxidase inhibition by daily oral administration of allopurinol (20 mg.kg** ^**−1**^ **, last 4 weeks) on high fructose high fat diet- induced insulin resistance (10**% **fructose in drinking water plus 25**% **unsaturated fat in diet, for 12 weeks) associated changes in body weight gain and serum levels of glucose, insulin and fructosamine and triglycerides and the insulin resistance (IR) index**

**Treatment**	**Body weight gain (%)**	**Glucose (mg.dl** ^**−1**^ **)**	**Fructosamine (mg.dl** ^**−1**^ **)**	**Insulin (μg.l** ^**−1**^ **)**	**Triglycerides (mg/dl)**	**IR index**
Control	32.46 ± 5.53	72.9 ± 2.68	46.4 ± 3.26	0.77 ± 0.11	48.41 ± 2.55	3.2 ± 0.44
IR	142.2 ± 29.18^**^	107.8 ± 5.06^***^	69.0 ± 5.67^**^	3.81 ± 0.7^*^	71.97 ± 13.17^*^	19.28 ± 3.29^**^
IR-Allo	115.5 ± 16.6	98.0 ± 3.78	62.2 ± 1.83	3.76 ± 0.85	47.49 ± 4.02^#^	21.87 ± 5.41

Values are expressed as the mean ± S.E of mean; N = 6-8 animals; ^*^P < 0.05, ^**^P < 0.01, ^***^P < 0.001, compared with the corresponding control group values; ^#^P < 0.05, compared with the corresponding insulin resistance group values by One Way ANOVA and Newman Keuls *post hoc* test.

### Basal cardiac hemodynamics

**Figure 2:**
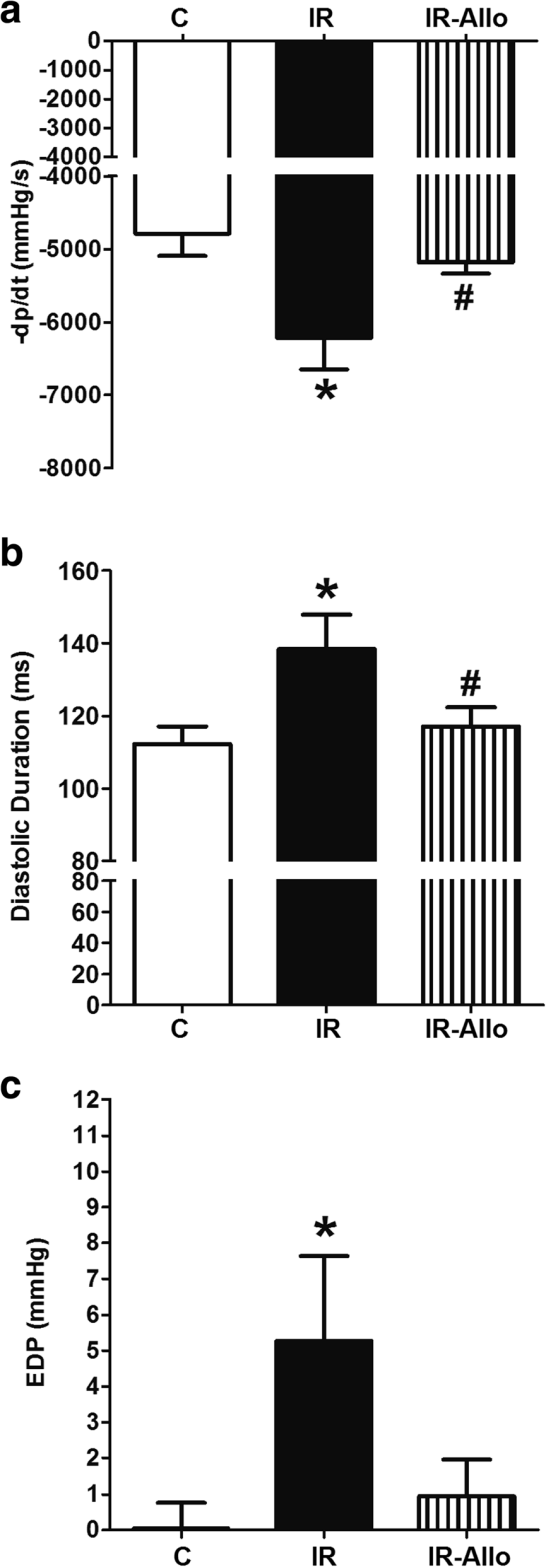
**Effect of xanthine oxidase inhibition by daily oral administration of allopurinol (20 mg.kg** ^**−1**^ **, last 4 weeks) on the slope of cardiac diastolic pressure decrement (a), diastolic duration (b) and end diastolic pressure (c) in rats with high fructose high fat (for 12 weeks) -induced insulin resistance (IR).** Data are presented as mean ± standard error of 8 animals in each group. ^*^P < 0.05, compared with the corresponding control group values; ^#^P < 0.05 compared with the corresponding IR group values; by one way ANOVA and Newman Keuls’ *post hoc* test.

IR induced by high fructose high fat diet resulted in a left ventricular diastolic dysfunction, reflected by a significant decrease in -dP/dt (p < 0.05, Figure 2a), significant increase in diastolic duration (p < 0.05, Figure 2b) and a significant increase in EDP (p < 0.05, Figure 2c) compared to the control group. XO inhibition by allopurinol prevented the left ventricular diastolic dysfunction associated with IR as indicated by the significant increase in -dP/dt and decrease in diastolic duration (both at p < 0.05, Figure 2a and b) compared with the IR group.

IR animals did not show significant changes in cardiac contractility index, heart rate or cycle duration compared with control animals (Table 2) although XO inhibition by allopurinol tended to increase the contractility index and decrease the heart rate compared with IR animals. IR significantly increased ESP (p < 0.01, Table 3) compared to control while XO inhibition did not affect this increase in ESP. Neither IR nor allopurinol affected the systolic duration or dP/dt significantly (Table 2).

**Table 2:** **Effect of xanthine oxidase inhibition by daily oral administration of allopurinol (20 mg.kg** ^**−1**^ **, last 4 weeks) on high fructose high fat diet- induced insulin resistance (10**% **fructose in drinking water plus 25**% **unsaturated fat in diet, for 12 weeks) associated changes in left ventricle contractility index, heart rate, cycle duration, end systolic pressure (ESP), systolic duration and maximum pressure development (dp/dt)**

**Treatment**	**Contractility index (1/s)**	**Heart rate (BPM)**	**Cycle duration (ms)**	**ESP (mmHg)**	**Systolic duration (s)**	**dp/dt (mmHg/s)**
Control	122.8 ± 10.88	289.4 ± 17.3	193.9 ± 6.32	87.48 ± 2.8	81.71 ± 1.79	5497 ± 626.3
IR	129.0 ± 18.21	284.5 ± 21.0	208.9 ± 13.19	109.8 ± 5.16^**^	80.8 ± 3.56	7428 ± 1167
IR-Allo	169.1 ± 14.6	250.4 ± 6.3	212.3 ± 9.26	110.5 ± 8.55	85.24 ± 2.92	7334 ± 720

Values are expressed as the mean ± S.E of mean; N = 6-8 animals; ^**^P < 0.01, compared with the corresponding control group values; by One Way ANOVA and Newman Keuls *post hoc* test.

**Table 3:** **Effect of xanthine oxidase inhibition by daily oral administration of allopurinol (20 mg.kg** ^**−1**^ **, last 4 weeks) on high fructose high fat diet- induced insulin resistance (10**% **fructose in drinking water plus 25**% **unsaturated fat in diet, for 12 weeks) associated changes in P, Q, R, S and T amplitudes**

**Treatment**	**P-amplitude (mV)**	**Q-amplitude (mV)**	**R-amplitude (mV)**	**S-amplitude (mV)**	**T-amplitude (mV)**
Control	−0.011 ± 0.015	−0.134 ± 0.063	0.346 ± 0.077	−0.17 ± 0.07	0.053 ± 0.035
IR	0.057 ± 0.025	−0.18 ± 0.136	0.67 ± 0.148	−0.42 ± 0.21	0.14 ± 0.08
IR-Allo	0.041 ± 0.02	−0.005 ± 0.009	0.527 ± 0.085	−0.28 ± 0.07	0.155 ± 0.046

Values are expressed as the mean ± S.E of mean; N = 6-8 animals; No significant differences were detected by One Way ANOVA.

### Basal cardiac ECG

**Figure 3:**
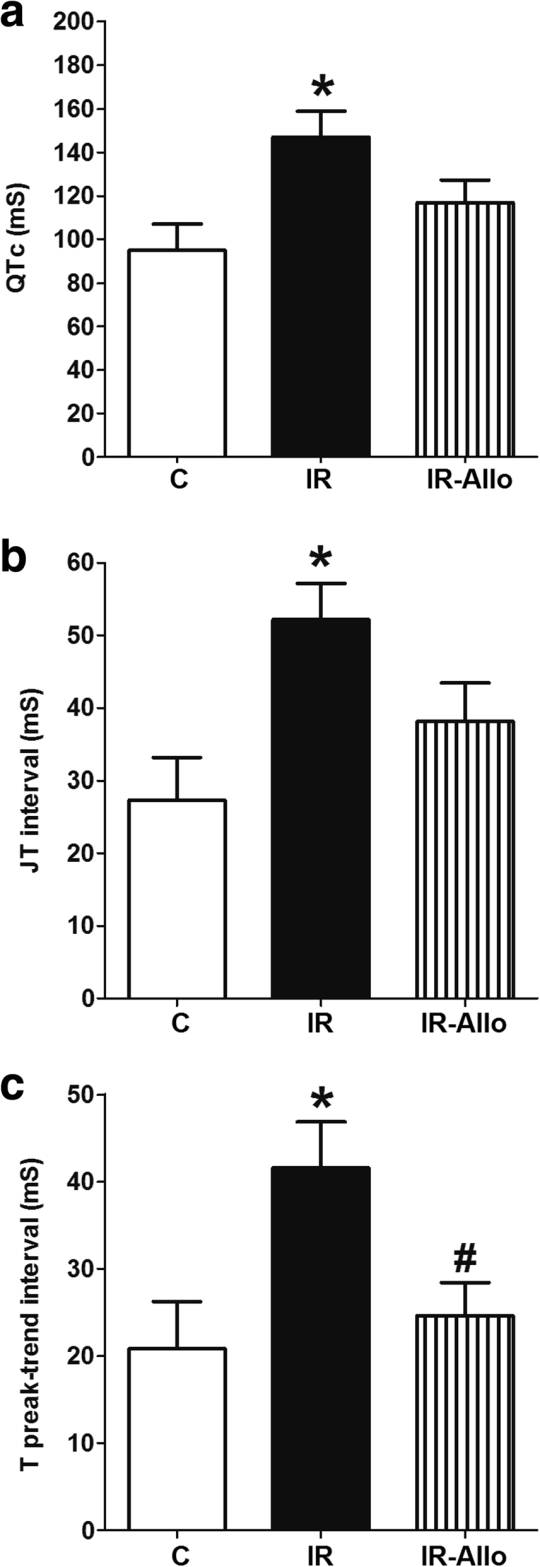
**Effect of xanthine oxidase inhibition by daily oral administration of allopurinol (20 mg.kg** ^**−1**^ **, last 4 weeks) on the slope of electrocardiograph QT (a), JT (b) and T peak trend (c) intervals in rats with high fructose high fat (for 12 weeks) induced insulin resistance (IR).** Data are presented as mean ± standard error of 8 animals in each group. ^*^P < 0.05, compared with the corresponding control group values; ^#^P < 0.05compared with the corresponding IR group values; by one way ANOVA and Newman Keuls’ *post hoc* test.

IR induced by high fructose high fat diet resulted in a prolongation of cardiac repolarization, reflected by a significant prolongation in QT, JT and T peak trend intervals compared to control (all at p < 0.05, Figure 3). XO inhibition by allopurinol alleviated the prolonged cardiac repolarization associated with IR as indicated by the significant decrease in T peak interval (p < 0.05) and that the QTc and JT was no longer significantly increased compared with control (Figure 3). Neither IR nor allopurinol affected the P, Q, R, S or T amplitudes significantly (Table 3).

### Isoproterenol-induced acute cardiac ischemia

**Figure 4:**
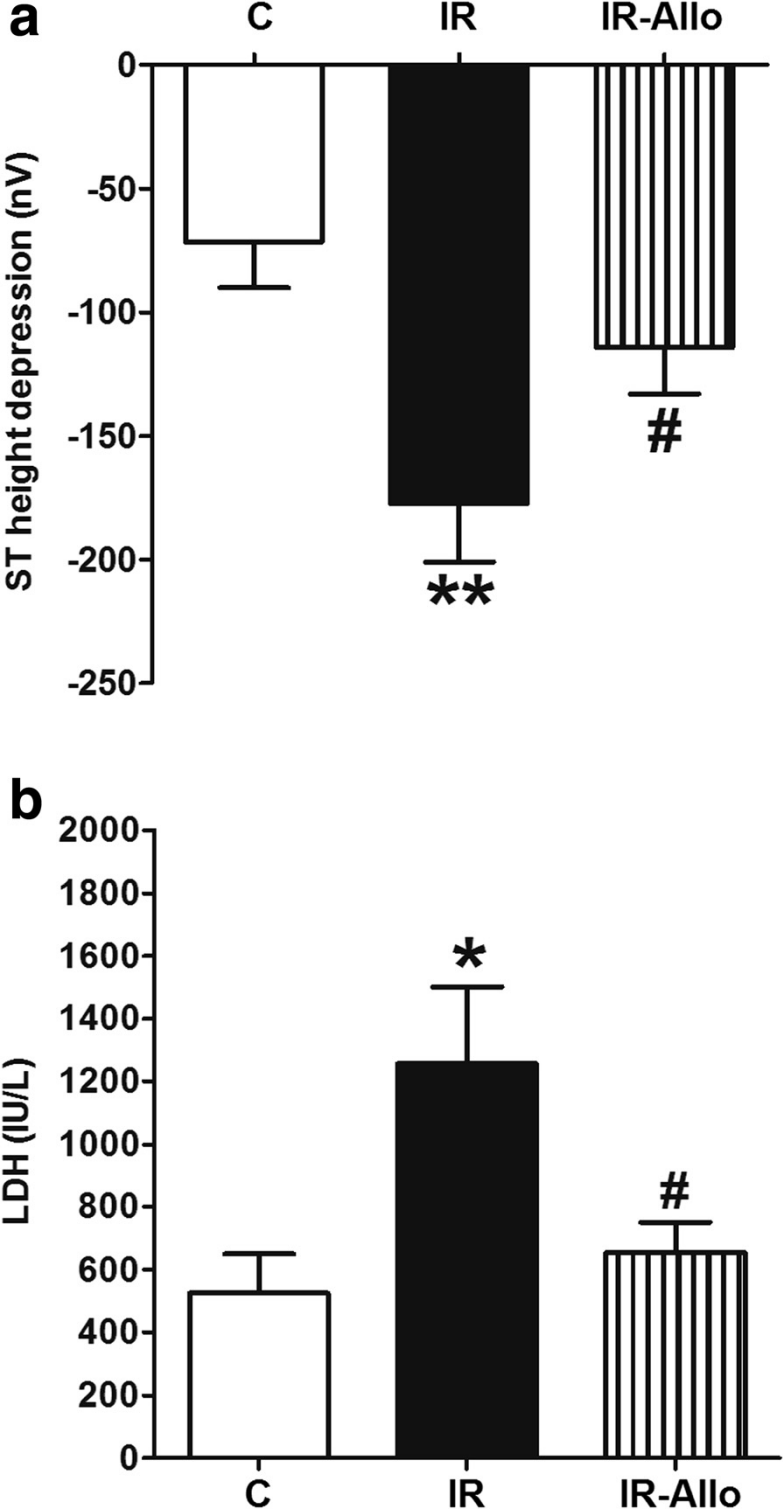
**Effect of xanthine oxidase inhibition by daily oral administration of allopurinol (20 mg.kg** ^**−1**^ **, last 4 weeks) on the ST height depression (a) and serum lactate dehydrogenase level (b) following isoproterenol-induced acute cardiac ischemia in rats with high fructose high fat (for 12 weeks) induced insulin resistance (IR).** Data are presented as mean ± standard error of 8 animals in each group. ^*^P < 0.05, ^**^P < 0.01, compared with the corresponding control group values; ^#^P < 0.05 compared with the corresponding IR group values; by one way ANOVA and Newman Keuls’ *post hoc* test.

Intravenous injection of isoproterenol led to induction of acute cardiac ischemia in anaesthetized rats as indicated by ST height depression and serum lactate dehydrogenase level elevation. Animals with IR were less resistant to acute cardiac ischemia and were characterized by greater cardiac ischemia as indicated by the significantly larger depression in ST height (p < 0.01, Figure 4a) and higher serum LDH activity (p < 0.05, Figure 4b) compared with control.

XO inhibition by allopurinol significantly alleviated the developed acute ischemia in hearts of IR animals as indicated by the significant reversal of ST height depression and LDH overactivity compared with the IR group (all at p < 0.05, Figure 4).

### Low grade inflammation

**Figure 5:**
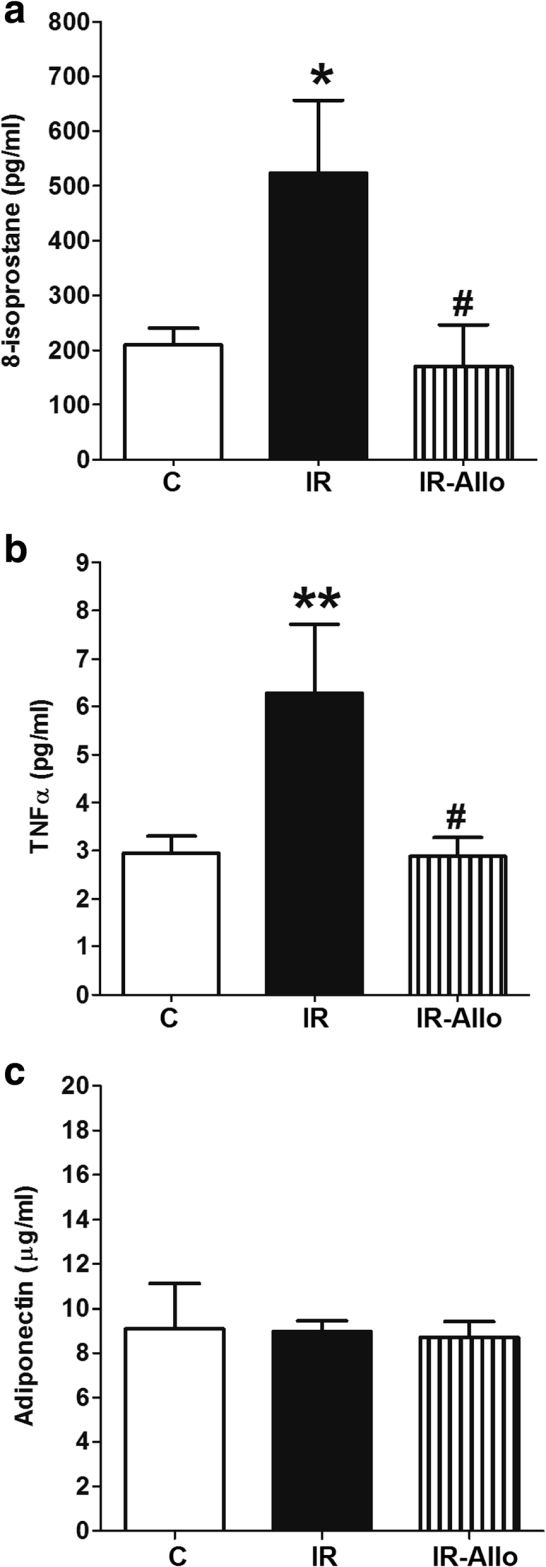
**Effect of xanthine oxidase inhibition by daily oral administration of allopurinol (20 mg.kg** ^**−1**^ **, last 4 weeks) on serum levels of TNFα (a), 8-isoprostane (b) and adiponectin (c) in rats with high fructose high fat (for 12 weeks) induced insulin resistance (IR).** Data are presented as mean ± standard error of 8 animals in each group. ^*^P < 0.05, compared with the corresponding control group values; ^#^P < 0.05 compared with the corresponding IR group values; by one way ANOVA and Newman Keuls’ *post hoc* test.

IR induced by high fructose high fat diet was associated with low grade inflammation and oxidative stress, reflected by significantly higher serum levels of the oxidative stress marker 8-isoprostane (p < 0.05, Figure 5a) and the inflammatory cytokine TNFα (p < 0.01, Figure 5b) compared to control. XO inhibition by allopurinol alleviated the low grade inflammation associated with IR as indicated by the significantly lower serum levels of 8-isoprostane and TNFα (both at p < 0.05) compared with IR group. Neither IR nor allopurinol affected the serum levels of the anti-inflammatory cytokine adiponectin significantly (Figure 5c).

### Cardiac oxidative stress and angiotensin system

**Figure 6:**
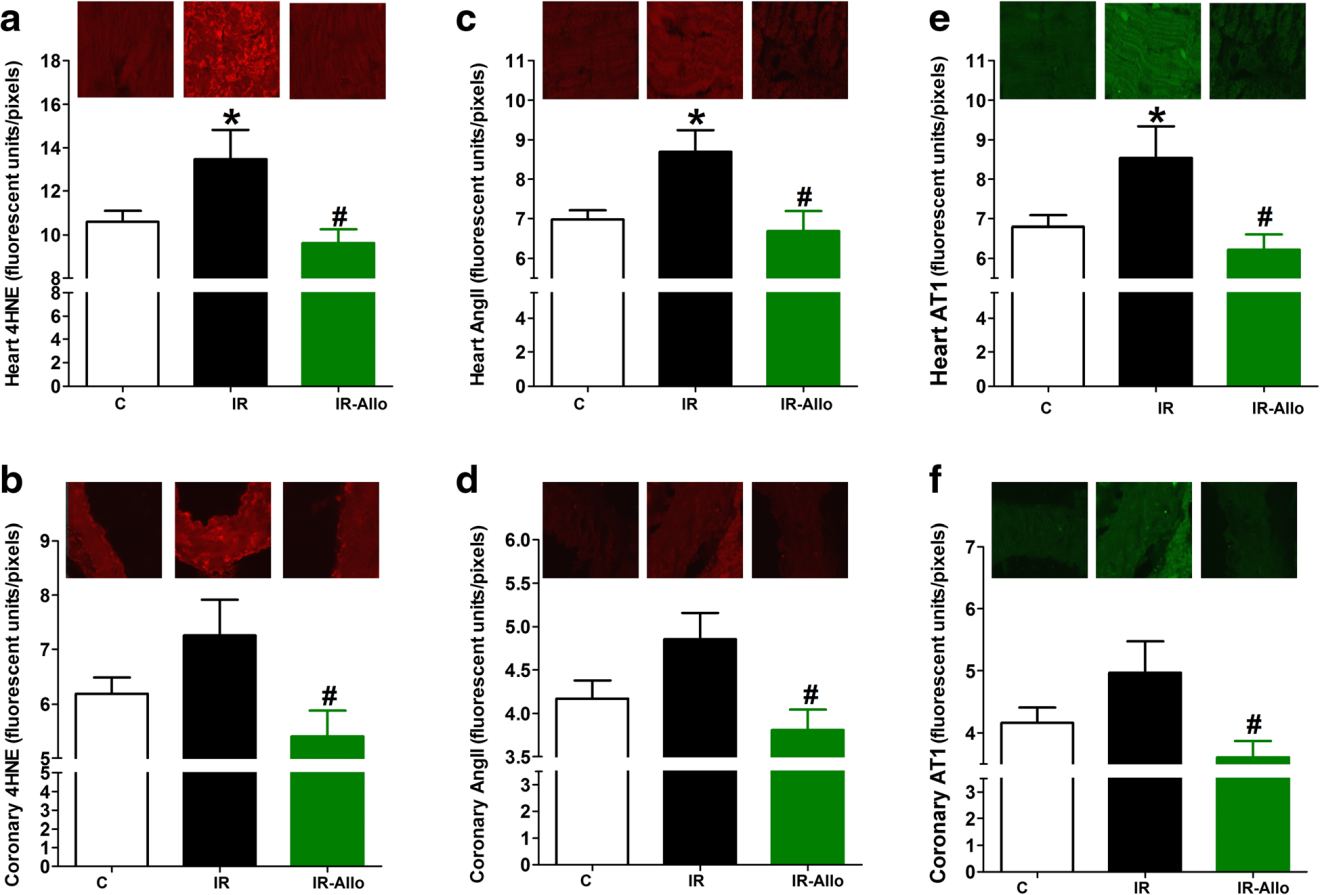
**Effect of xanthine oxidase inhibition by daily oral administration of allopurinol (20 mg.kg** ^**−1**^ **, last 4 weeks) on 4-hydroxy-2-noneal Michael Adducts (4HNE), angiotensin II (AngII) and angiotensin receptor type 1 (AT**_**1**_ **) immunofluorescence in heart (a, c and e respectively) and coronary artery (b, d and f respectively) sections from rats with high fructose high fat (for 12 weeks) induced insulin resistance (IR).** Data are presented as mean ± standard error of 8 animals in each group. ^*^P < 0.05, compared with the corresponding control group values; ^#^P < 0.05 compared with the corresponding IR group values; by one way ANOVA and Newman Keuls’ *post hoc* test. Micrographs at the top are representative fluorescence images of heart and coronary artery cross sections immunofluorescence stained by 4HNE, AngII or AT_1_ antibodies followed by Alexa fluor conjugated secondary antibodies.

Heart cross sections from animals with IR showed a significant increase immunofluorescence for the lipid peroxidation product 4HNE (p < 0.05, Figure 6a) compared with control. XO inhibition by allopurinol completely blocked the increase in 4HNE immunofluorescence both in heart tissue and coronary artery (both at p < 0.05, Figure 6a and b). In addition, IR was associated with activation of the angiotensin system as indicated by the significant increase in immunofluorescence for AngII and its receptor AT_1_ (Figure 6c and e, both at P < 0.05). Allopurinol completely prevented the increase in immunofluorescence of AngII and AT_1_ both in heart tissue and coronary artery from IR animals (Figure 6c-f).

## Discussion

The current study is the first to report on the protective effect of XO inhibition against the cardiac diastolic dysfunction and ischemia associated with IR. The XO inhibitor allopurinol virtually abolished the development of cardiac diastolic dysfunction and ischemia in high fructose high fat diet-induced IR in rats without affecting the developed hyperinsulinemia or dyslipidemia. The following findings support the conclusion that XO inhibition can counteract cardiac diastolic dysfunction and ischemia accompanying IR: (i) XO inhibition ameliorated the impaired left ventricular diastolic relaxation associated with IR; (ii) the prolongation of cardiac repolarization in IR was alleviated by XO inhibition; (iii) XO inhibition alleviated the exaggerated cardiac ischemia following isoproterenol injection in IR; (iv) XO inhibition alleviated the low grade inflammation and oxidative stress associated with IR; (v) XO inhibition blocked the IR-induced activation of the angiotensin system both in heart tissue and coronary artery. These findings provide convincing evidence that XO inhibition offsets the cardiac diastolic dysfunction and ischemia accompanying IR in addition to the associated low grade inflammation and oxidative stress.

The predominant feature in cardiomyopathy associated with diabetes, metabolic syndrome and IR seems to be the left ventricular diastolic dysfunction as shown in the present study and indicated in previous works [18,19]. Ventricular relaxation is considered as an active process that depends mainly on the intracellular calcium uptake by the endoplasmic reticulum during diastole [20]. We reasoned that XO inhibition would minimize the cardiac dysfunction associated with IR. The validity of this assumption was ascertained in this work because allopurinol, the standard XO inhibitor, prevented the left ventricular diastolic dysfunction associated with IR as indicated by the significant increase in -dP/dt and decrease in diastolic duration compared with the IR group. In agreement with this, XO activation has been shown to contribute to left ventricular diastolic dysfunction in an experimental model of cocaine-induced diastolic dysfunction [21].

Prolongation of cardiac repolarization is an important cardiovascular risk factor in several diseases, including diabetes mellitus and infarction [22]. Animals with IR in this study have shown prolonged cardiac repolarization reflected by the significant increase in QTc, JT and T peak trend intervals compared to control. The role of prolonged repolarization in type II diabetes is not well established, although repolarization heterogeneity is observed [23]. In our work we demonstrate normalization of the repolarization by allopurinol, implicating xanthine oxidase-derived ROS. The molecular mechanism for prolonged repolarization in IR is not clear, but appears to involve ROS-dependent oxidation and activation of p90 ribosomal S6 kinase and inhibition of voltage gated K^+^ channels [24], and/or Ca/calmodulin-dependent protein kinase II and cardiac sodium channels [25].

The results of the present study show that animals with IR are less resistant to isoproterenol-induced acute cardiac ischemia as indicated by the significantly larger depression in ST height and higher serum LDH activity compared with control. This is in agreement with clinical literature where MetS is associated with a high incidence of coronary atheroma and an increased risk of fatal coronary events [26]. The fructose-induced model of MetS was associated with clear arterial atheroma in previous work of our laboratories [17]. In the present study, XO inhibition by allopurinol significantly alleviated the developed ischemia in hearts of IR animals as indicated by the significant decrease in ST height depression and LDH activity compared with animals not receiving allopurinol. This is in accordance with the reported anti-ischemic activities for allopurinol in patients with ischemic heart disease [27].

The protective effect of XO inhibition against cardiac diastolic dysfunction and ischemia in animals with IR seems to be direct cardiovascular protection rather than affecting IR itself, because XO inhibition did not have a significant effect on the IR index. However, it did significantly alleviate the cardiac diastolic dysfunction and ischemia in animals with IR. This also provides proof for the assumption that hyperuricemia and XO activation are independent risk factors for cardiovascular disease in IR. Numerous current epidemiological studies point to a possible role of uric acid as an independent risk factor for cardiovascular complications in metabolic syndrome, but without definitive conclusions [4].

The mechanism(s) by which XO inhibition alleviates cardiac diastolic dysfunction and ischemia in animals with IR could be by a reduction of the associated hyperuricemia and oxidative stress. Although uric acid has antioxidant effects extracellularly, it stimulates inflammatory and oxidative stress mechanisms inside the cell [28]. This results in an inhibition of endothelial dependent relaxation and nitric oxide (NO) production [29]. We have previously shown that *in vitro* incubation with uric acid at the concentration observed in MetS animals impaired endothelial relaxation in isolated normal aorta [5]. In the present study, we found that allopurinol completely prevented hyperuricemia in animals with IR.

Oxidative stress and low-grade inflammation are clearly involved in pathogenesis of the IR and MetS and its complications [30]. The reactive oxygen species (ROS) generated by enzymes including the XO in IR and MetS interact with various cellular proteins resulting in inactivation and loss of function [31]. In the cardiovascular system, ROS can impair relaxation by mopping up of released NO, uncoupling of endothelial NO synthase and activating transcription of inflammatory genes [32]. We have previously shown elevated aortic ROS [33], nuclear factor kappa B activation and serum TNFα [17] in animal models of metabolic syndrome that were significantly correlated with impaired NO production and endothelial dependent relaxation [34]. In the present study, animals with IR had significantly higher serum levels of the oxidative stress marker 8-isoprostane and TNFα while XO inhibition by allopurinol completely prevented these elevations. In addition, XO inhibition completely blocked increases in the lipid peroxidation product 4HNE both in heart tissue and coronary artery. This is consistent with previous work where allopurinol alleviated the elevated liver malondialdehyde and TNFα associated with acute liver failure induced by thioacetamide in rats [35].

The angiotensin system is one of the most important oxidative stress systems activated in MetS. The major effector peptide in the system is AngII, which binds to AT_1_ or AT_2_ receptors to exert different biological functions. Binding of AngII to AT_1_ has been shown to produce the second messengers inositol trisphosphate and diacylglycerol which lead to stimulation of reactive oxygen species and vasoconstriction in numerous previous reports (reviewed by Frigolet *et al.* [36]). In the present work, XO inhibition completely prevented the increase in AngII and AT_1_ in both heart tissue and coronary artery from IR animals. This is in accordance with a previous study showing that allopurinol attenuated oxidative stress and cardiac dysfunction induced by AngII infusion in mice [37].

The possibility that modulation in serum lipids contributed to IR-XO cardiac dysfunction was also examined. Dyslipidemia is associated with increased cardiovascular mortality and morbidity [38]. Our findings showed that IR was characterized by hypertriglyceridemia while XO inhibition completely prevented hypertriglyceridemia associated with IR. This is in accordance with previous reports where allopurinol inhibited the hypertriglyceridemia in diabetic animals [39]. These observations suggest that the cardioprotective effect of XO inhibition may be mediated, at least in part, by amelioration of hypertriglyceridemia.

## Conclusions

XO inhibition by allopurinol alleviates cardiac diastolic dysfunction and ischemia associated with IR. Inhibition of hyperuricemia, low grade inflammation and the angiotensin system are suggested potential mechanisms for the observed cardiac protection. It remains to be determined whether the cardiac dysfunction observed is the consequence of one cytokine or of the combinatorial effects of multiple mediators, and the effect of blocking individual cytokines in this model may provide further mechanistic insight. The findings from this study suggest that xanthine oxidase may provide a novel drug target for the treatment of cardiac complications associated with insulin resistance and diabetes.

## Abbreviations

4HNE: 4-Hydroxy-2-noneal Michael Adducts
ARRIVE: Animals in Research: Reporting In Vivo Experiments
ANOVA: Analysis of variance
AngII: Angiotensin II
AT_1_: Angiotensin receptor type 1
PBS: Bovine serum albumin
ECG: Surface electrocardiography
EDP: Left ventricular end-diastolic pressure
ESP: End ventricular systolic pressure
LDH: Lactate dehydrogenase
MetS: Metabolic syndrome
NO: Nitric oxide
PBS: Phosphate buffered saline
ROS: Reactive oxygen species
dP/dt: Slope of systolic pressure increment
−dP/dt: Slope of diastolic pressure decrement
TNFα: Tumor necrosis factor alpha
XO: Xanthine oxidase
